# Bisphenol A Binds to the Local Anesthetic Receptor Site to Block the Human Cardiac Sodium Channel

**DOI:** 10.1371/journal.pone.0041667

**Published:** 2012-07-27

**Authors:** Andrias O. O’Reilly, Esther Eberhardt, Christian Weidner, Christian Alzheimer, B. A. Wallace, Angelika Lampert

**Affiliations:** 1 Institute of Physiology and Pathophysiology, Friedrich-Alexander-Universität Erlangen-Nürnberg, Bavaria, Germany; 2 Department of Crystallography, Institute of Structural and Molecular Biology, Birkbeck College, University of London, London, United Kingdom; 3 Bavarian Health and Food Safety Authority, Erlangen, Germany; Georgia State University, United States of America

## Abstract

Bisphenol A (BPA) has attracted considerable public attention as it leaches from plastic used in food containers, is detectable in human fluids and recent epidemiologic studies link BPA exposure with diseases including cardiovascular disorders. As heart-toxicity may derive from modified cardiac electrophysiology, we investigated the interaction between BPA and hNav1.5, the predominant voltage-gated sodium channel subtype expressed in the human heart. Electrophysiology studies of heterologously-expressed hNav1.5 determined that BPA blocks the channel with a K_d_ of 25.4±1.3 µM. By comparing the effects of BPA and the local anesthetic mexiletine on wild type hNav1.5 and the F1760A mutant, we demonstrate that both compounds share an overlapping binding site. With a key binding determinant thus identified, an homology model of hNav1.5 was generated based on the recently-reported crystal structure of the bacterial voltage-gated sodium channel NavAb. Docking predictions position both ligands in a cavity delimited by F1760 and contiguous with the DIII–IV pore fenestration. Steered molecular dynamics simulations used to assess routes of ligand ingress indicate that the DIII–IV pore fenestration is a viable access pathway. Therefore BPA block of the human heart sodium channel involves the local anesthetic receptor and both BPA and mexiletine may enter the closed-state pore via membrane-located side fenestrations.

## Introduction

Bisphenol A (BPA) is used abundantly in the manufacture of polycarbonate plastics and epoxy resins. Millions of tons of BPA are produced annually and it is detectable in the urine and blood of a large section of the population [1], [2], . Given the short half-life (<6 hours) of BPA in the body [4], its frequent detection indicates that there is pervasive environmental exposure to the compound. One entry route to the body is ingestion when leached from the lining of food and beverage containers [5].

Animal studies have linked BPA exposure to changes in brain and behavior, prostate and breast cancer, miscarriage, birth defects, diabetes and obesity [6], raising issues concerning BPA safety limits. Two studies have shown a correlation between urine concentration of BPA and heart disease in human populations [7], [8]. While BPA has been characterized as an endocrine disruptor, displaying weak agonistic effects at estrogenic receptors [9], [10], the broad spectrum of suspected BPA-associated diseases, including disorders of the heart, suggests additional molecular targets may exist.

The electrical excitability of cardiac myocytes is mediated by ion channels, with the activity of the heart-specific voltage-gated sodium channel isotype hNav1.5 generating the upstroke of the action potential. The critical role hNav1.5 plays in cardiac health is evident from the number of pro-arrhythmogenic channelopathies associated with mutation of the SCN5A gene encoding hNav1.5, which include long QT syndrome type 3, Brugada syndrome and sudden infant death syndrome [11]. Conversely, cardiac dysrhythmias can be treated by modulating hNav1.5 activity using 1A and 1B antiarrhythmics, which are members of the local anesthetic (LA) class of channel-blocking drugs.

LAs bind to closed-resting states of voltage-gated sodium channels (Na_v_s) but display greater affinity (>100-fold) for open or open-inactivated states [12]. As such, their inhibition is most potent when channels repetitively open and inactivate during sustained periods of action potential firings that characterize, for example, cardiac tachy-arrhythmias. The modulated receptor hypothesis [13] was proposed to account for the state-dependent difference in LA affinity, whereby the high-affinity binding site is formed in a ‘use-’ or ‘frequency-dependent’ manner during the conformational shift from closed-resting to activated or inactivated states. LAs also block closed-state Na_v_ to produce ‘resting state’ or ‘tonic’ block, albeit with lower affinity [12].

The receptor site for LAs has been localized to the transmembrane pore region of Na_v_, which is formed when the four pseudo-homologous domains (DI–DIV) of the Na_v_ α-subunit assemble to form a central ion-conducting pathway. Each domain is comprised of six transmembrane helices (S1–S6): S1–S4 form the channel voltage-sensors, S5 and S6 the pore module and the extracellular S5–S6 linkers (termed the P-loops) create the selectivity filter. A number of residues on the DIII and DIV S6 helices contribute to the LA binding site, with the aromatic residue F1760 forming the critical binding determinant; both use-dependent and tonic block is attenuated with mutation of this residue [14], [15].

The first crystal structure of a Na_v_ has recently been reported [16]: NavAb from the bacterium *Arcobacter butzleri* is a homo-tetrameric channel. Although its voltage-sensors apparently adopt an activated-state conformation, the pore domain is tightly constricted at the cytoplasmic end and therefore adopts a closed-state conformation. While providing valuable insights into Na_v_ structural elements such as the selectivity filter, of relevance for ligand studies is the presence of fenestrations of dimensions 8×10 Å within the transmembrane region of the pore domain; they appear to connect the lipid bilayer to the pore lumen. Payandeh et al. hypothesized that equivalent fenestrations in eukaryotic Na_v_ channels may provide accessible pathways for pore-blocking LAs. Indeed, the fenestrations of NavAb are occupied by the acyl chains of co-crystallized lipid molecules, thus demonstrating that it is feasible for hydrophobic exogenous molecules to traverse these fenestrations.

Here we show with whole cell patch-clamp recordings that currents through hNav1.5 expressed in HEK cells are blocked by micromolar concentrations of BPA, displaying a tonic and use-dependent component. Mutagenesis of F1760 to alanine reduced the BPA-induced tonic and use-dependent block, indicating that the binding site of BPA overlaps with that of local anesthetics. Steered molecular dynamics simulations using a hNav1.5 homology model indicate that BPA and mexiletine can feasibly access the closed-state pore via the side fenestrations and thus bind in the local anesthetic receptor site.

## Materials and Methods

### Cell Culture and Mutagenesis

The plasmid DNA of hNav1.5 in pTracer was used (gift from Dirk Isbrandt, Hamburg, Germany) for mutagenesis of F1760A (Qickchange XL kit, Qiagen GmbH, Hilden) and was performed according to the instructions of the manufacturer. HEK293 cells were maintained in Dulbecco’s Modified Eagle’s Medium (Invitrogen, Carlsbad, CA), supplemented with 10% fetal bovine serum, 100 U/ml penicillin and 100 µg/ml streptomycin, and incubated at 37°C with 5% CO_2_. Briefly, HEK293 cells were plated at low density on 35 mm dishes 24 hours before transfection. Cells were transfected at 40–70% confluency using Nanofectin (PAA Laboratories GmbH, Pasching, Austria), according to manufacturer’s instructions, with 1 µg of hNav1.5 or the F1760A mutant of hNav1.5 and 0.5 µg EGFP, and distributed onto several 35 mm dishes on the following day. Electrophysiology was performed 18–28 hours following transfections. Only cells with robust green fluorescence were included in the analysis.

### Electrophysiology

Whole-cell patch-clamp experiments were performed at room temperature using an Axopatch 200A operated by Clampex 9.2 software (Axon Instruments, Molecular Devices, USA) and 1–2 MΩ glass electrodes (WPI Inc, USA). The pipette solution contained (in mM): 140 CsF, 10 NaCl, 1 EGTA, and 10 HEPES; 302 mosmol (pH 7.4, adjusted with CsOH) and the extracellular bath contained (in mM): 140 NaCl, 3 KCl, 10 glucose, 10 HEPES, 1 MgCl_2_, 1 CaCl_2_; 310 mosmol (pH 7.4, adjusted with NaOH). All reagents were purchased from Sigma Chemical Company (Deisenhofen, Germany) unless otherwise noted. The tip of a constantly running gravity-driven perfusion system was positioned close to the recorded cell and drugs were applied by switching to bath solution containing the indicated concentration. BPA was dissolved in ethanol at a stock concentration of 100 mM and diluted, reaching a maximum final ethanol concentration of 0.3%, whereas lidocaine, lamotrigine and mexiletine were directly added to the bath solution. Capacitive transients were cancelled, and series resistances (<5 MΩ) were compensated by 65–95%. Leak currents were subtracted digitally online using the P/4 procedure following the test pulses except for recordings of use dependency, where no leak correction was used. Currents were filtered at 10 kHz and sampled at 100 kHz, except for steady-state fast inactivation, where a sampling rate of 20 kHz was used.Recordings were obtained 4 min after establishing the whole-cell configuration. The drugs or 0.1% ETHO were applied after pulse 10 at the indicated concentrations during a series of test pulses to −10 mV for 250 ms every 5 s from a holding potential of −120 mV. The block occurring 15 pulses after BPA or ETOH application (I_post_/I_pre_  = 
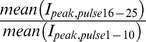
) was used as indicator for tonic block. The resulting single dose response data were fitted with a Hill function using a Levenberg-Marquard algorithm of Origin’s non linear curve fitter, where start and end were set to 0 and 1, respectively. Fitting parameters (K_d_) are given as best fit ± standard error.

Data analyses were performed using Clampfit 9.2 (Axon Instruments, Molecular Devices, USA), Excel (Microsoft Corporation, USA), Origin 7 (OriginLab Corporation, USA), and Statistica (StatSoft (Europe), Germany). The statistical significances of differences between mean(I_peak,pulse1–10_) and mean(I_peak,pulse16–25_) were assessed using a paired one-sided Student’s t-test and was set at p<0.05. For values of the dose-response curves of WT and the F1760A mutant of hNav1.5 and comparison of their use-dependent block, ANOVA with posthoc Fischer LSD analysis was used. All data are presented as mean ± SEM, unless noted otherwise.

Current-voltage (I–V) relations were obtained using 250 ms pulses from a holding potential of −120 mV to a range of test potentials (−90 to +45 mV) in 10 mV steps with 5 seconds between pulses. The sodium channel conductance G_Na_ was calculated using the following equation:


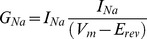


where I_Na_ is the amplitude of the current at the voltage V_m_, and E_rev_ is the reversal potential for sodium, which was determined for each cell individually. Activation curves were derived by plotting normalized G_Na_ as a function of test potential and fitted using the Boltzmann equation:


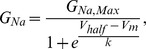


where G_Na,Max_ is the maximum conductance, V_half_ is the membrane potential at half-maximal activation, V_m_ is the membrane voltage and *k* is the slope factor.

To examine the voltage-dependences of steady-state fast inactivation, a series of 500 ms prepulses (−160 to 0 mV) from a V_hold_ of −120 mV, each followed by a brief test pulse to −10 mV, were presented at 5 sec intervals. Normalized current amplitude (I_Na_/I_Na,Max_) at each test potential was plotted as a function of prepulse potential (V_m_) and fitted using the following (Boltzmann) equation:
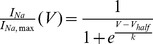
, yielding the half maximal inactivation (Vhalf) and the slope factor (k).

Decay time constants were determined by fitting current recordings at test pulses to −10 mV for 40 ms from peak with a single exponential fit.

The dissociation constant for the resting channels, K_r_, of the applied drugs were determined according to [17], [18]:


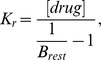


with [drug] being the drug concentration present and B_rest_ being the detected block of resting channels.

Dissociation constants for inactivated channels, K_i_, were determined by holding the recorded cells at a potential at which 30–50% of channels are inactivated. This potential was determined for each cell individually by the above described protocol for steady-state fast inactivation. Test pulses (250 ms) to −10 mV were applied every 5 sec and the drug was perfused. K_i_ was determined as [17], [18]:

K_i_  =  
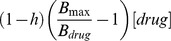


with *h* being the fraction of inactivated channels at the chosen holding potential, B_max_ being the maximal possible block by the drug, which was assumed to be complete and therefore set to 1 and B_drug_ is the amount of block at [drug].

With K_r_ and K_i_ the shift of V_half_ of steady-state fast inactivation can be predicted with the following equation [17], [18]:


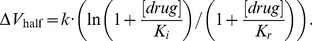


When two drugs, drug1 and drug2, are applied simultaneously the drug induced shift of V_half_ can be predicted for separate binding sites via:


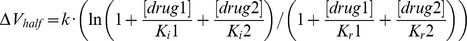


and for the same or an overlapping binding site via:


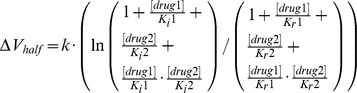


Use-dependent current decline was examined before and after drug application using 30 test pulses to −10 mV at frequencies of 1 Hz, 10 Hz (for both the test pulse length was 40 ms), 30 Hz (test pulse 20 ms) and 50 Hz (test pulse 10 ms) from a holding potential of −120 mV. Current responses were normalized to the first recorded pulse and the available currents at the 30^th^ pulses were compared.

### Homology Modeling and Automated Ligand Docking Studies

The 2.7 Å crystal structure of the bacterial sodium channel NavAb (PDB code 3RVY, [16]) was used as the structural template for construction of a closed-state hNav1.5 homology model. Sequences were aligned using ClustalW [19] and 50 models were produced using MODELLER [20]. The internal scoring function of MODELLER was used to select 10 models, which were visually inspected and submitted to the VADAR webserver [21] for assessment of stereochemical soundness. The lead candidate was energy-minimized for 20 picoseconds (ps) using NAMD molecular dynamics software (version 2.8) [22]. Energy minimization and molecular dynamics simulations (detailed below) were carried out *in vacuo* and, in order to closely preserve the initial structure of the channel in the absence of solvent molecules yet still allow a degree of conformational flexibility, a harmonic constraint of value 0.75 was applied to the model backbone atoms. The ‘solvate’ function of VMD was used to calculate how water molecules could be accommodated in the model pore and therefore assess its volume.

Crystal structures of BPA (reference code CEGYOC02) and mexiletine (reference code JIZJEH) were downloaded from the Cambridge Structural Database (www.ccdc.cam.ac.uk). Automated docking predictions of these ligands with the channel model were generated using Autodock 4 as previously described [23]. Grid maps with 60×60×60 points and 0.375 Å spacing were constructed to encompass the pore interior. Dockings were performed using a Lamarckian genetic algorithm [24] with population size = 150, mutation rate = 0.02, crossover rate = 0.8. Docking predictions were screened by interaction energy and proximity (≤4 Å) of the ligand to the side-chain of F1760. Figures were produced using PyMOL (DeLano Scientific, San Carlos, CA, U.S.A.).

### Steered Molecular Dynamics (SMD) Simulations

SMD simulations of BPA or mexiletine ingressing the closed-state hNa_v_1.5 pore were performed using NAMD [22]. Topology and parameter files were generated for the ligands using the SwissParam web-server (http://swissparam.ch). VMD software [25] and the PDBSET program of the CCP4 suite [26] were used to (a) position the hNa_v_1.5 model with the centroid at the pore center, (b) position either BPA or mexiletine 35 Å from the pore center along a coordinate axis that ran through either a pore fenestration or the selectivity filter, (c) rotate each positioned ligand (by changing Euler angles in 120° steps) to produce 21 starting models per fenestration or filter. This approach of using many starting orientations enabled the comparison of force calculations for different access routes as it circumvented the bias that could result if a single orientation was used for all simulations (since a ligand may more favourably traverse one pathway based on that particular orientation).

During simulations ligands were drawn towards the pore center by constant velocity pulling, whereby the ligand was attached by a virtual spring to a massless “dummy atom” moving at 4 Å/ps along a coordinate axis. The force (F) exerted on the ligand was:


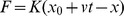


where K = 139 picoNewton/Å is the spring constant, v = 0.004 Å/time-step is the pulling velocity and *x* and *x_0_* are the positions of the ligand’s center-of-mass and the end of the spring, respectively. 20 pS simulations using the CHARMM22 protein force field were carried out at a temperature of 310 K with a 1 femtosecond time-step; electrostatic and van der Waals interactions were truncated at 12 Å. VMD was used for simulation analysis and extraction of the force exerted on the ligand center-of-mass in the direction of spring movement. The average values were plotted for the force for each set of fenestration or filter simulations.

## Results

### BPA Blocks hNav1.5 Currents

**Figure 1:**
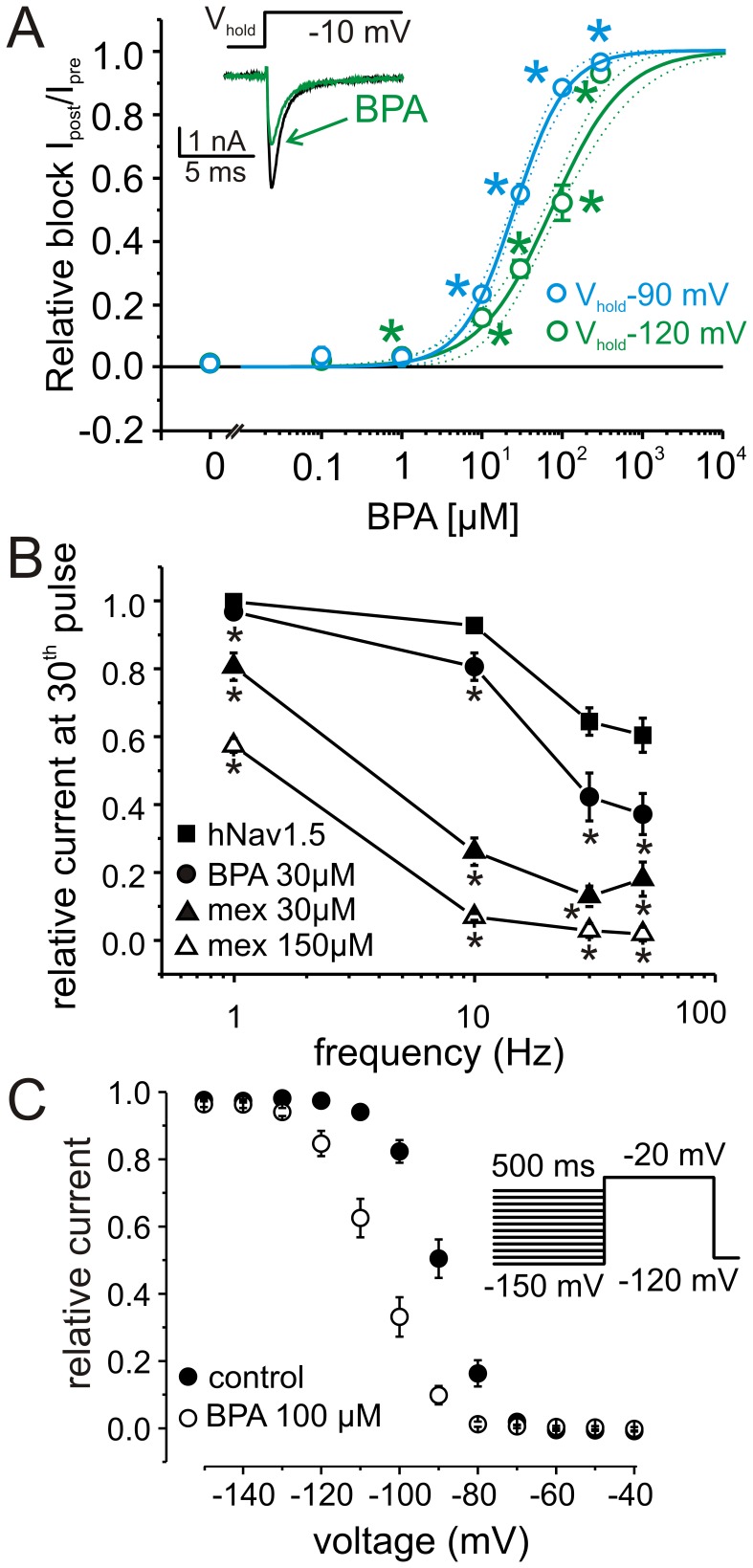
BPA is a blocker of human heart sodium channels (hNav1.5). A: Dose response curve of tonic block of hNav1.5 by BPA is shown as the relative decrease of sodium current response to a square voltage step to −10 mV (see inset) at various concentrations of BPA from a V_hold_ of −120 mV (green circles) or −90 mV (blue circles). A dose response function (solid lines, Hill function, K_d_ = 74.0±7.6 µM and 25.4±1.3 µM, for −120 and −90 mV, respectively) was employed and its 95% confidence intervals (dotted lines) were determined. n = 6−15 per point, * indicates significance in a paired one sided t-test. Value at 0 mM corresponds to a vehicle control with application of 0.1% ETOH. *Inset:* Representative current traces recorded from a HEK293t cell expressing hNav1.5 before and during application of 30 µM BPA. V_hold_ = −120 mV. B: Use-dependent block of currents through hNav1.5 expressed in HEK cells (squares) was enhanced by local anesthetics, such as mexiletine (30 µM, filled triangles, 150 µM, open triangles, p<0.001 for all frequencies). A similar enhancement was observed with BPA and is more pronounced at higher frequencies (30µM, filled circles). Test pulse potential was −10 mV, V_hold_ = −120 mV, and test pulse length varied from 10 to 40 ms, depending on the frequency. C: Steady-state fast inactivation of hNav1.5 before and after application of 100 µM BPA (n = 9). Cells were held at −120 mV and fast inactivation was induced by a 500 ms prepulse to the indicated potentials (see inset). Available channels were assessed by a step depolarization to −20 mV and resulting curves were fitted with a Boltzmann function (V_half_ = −89.3±1.5 mV, *k* = 5.6±0.3 pre; and V_half_ = −105.1±2.1 mV, *k* = 6.5±0.2 post application of 100 µM BPA).

To investigate the effects of BPA on cardiac sodium channel function, we transiently expressed hNav1.5 in HEK cells and performed whole-cell patch clamp experiments (Fig. 1). Tonic block of hNav1.5 by BPA was assessed with depolarizing test pulses applied at a frequency of 0.2 Hz, which did not induce use-dependent current decline. Application of micromolar concentrations of BPA induced a dose-dependent tonic block with a K_d_ of 74.0±7.6 µM (Hill fit, Fig. 1, green symbols, V_hold_ = −120 mV, n = 5−11). For cells held at more depolarized potentials closer to the physiological cardiac resting membrane potential (V_hold_ = −90 mV), this block was more pronounced (Fig. 1, blue symbols, K_d_ = 25.4±1.3 µM, n = 5−10).

At V_hold_ = −90 mV more hNav1.5 channels are in the inactivated state than at V_hold_ = −120 mV. Therefore the predominant block of BPA at this holding potential may result from a higher binding affinity of the compound to the inactivated state, which is the case for LAs, where dissociation constants for inactivated channels is much lower than for resting channels (K_i_<<K_r_). Indeed BPA did enhance use-dependent current decline at all frequencies tested (1 to 100 Hz, Fig. 1B), without affecting activation or the time constant of current decay (V_half_ of activation: −32.0±2.5 mV (pre) and −32.9±3.7 mV (post 100µM BPA), τ of single exponential fit of current decay: 1.1±0.08 ms (pre), 1.13±0.1 ms (post), test pulse to −10 mV, n = 4, 7).

### BPA Shifts Steady-state Fast Inactivation of hNav1.5 to More Hyperpolarized Potentials, Similar to LAs

**Figure 2:**
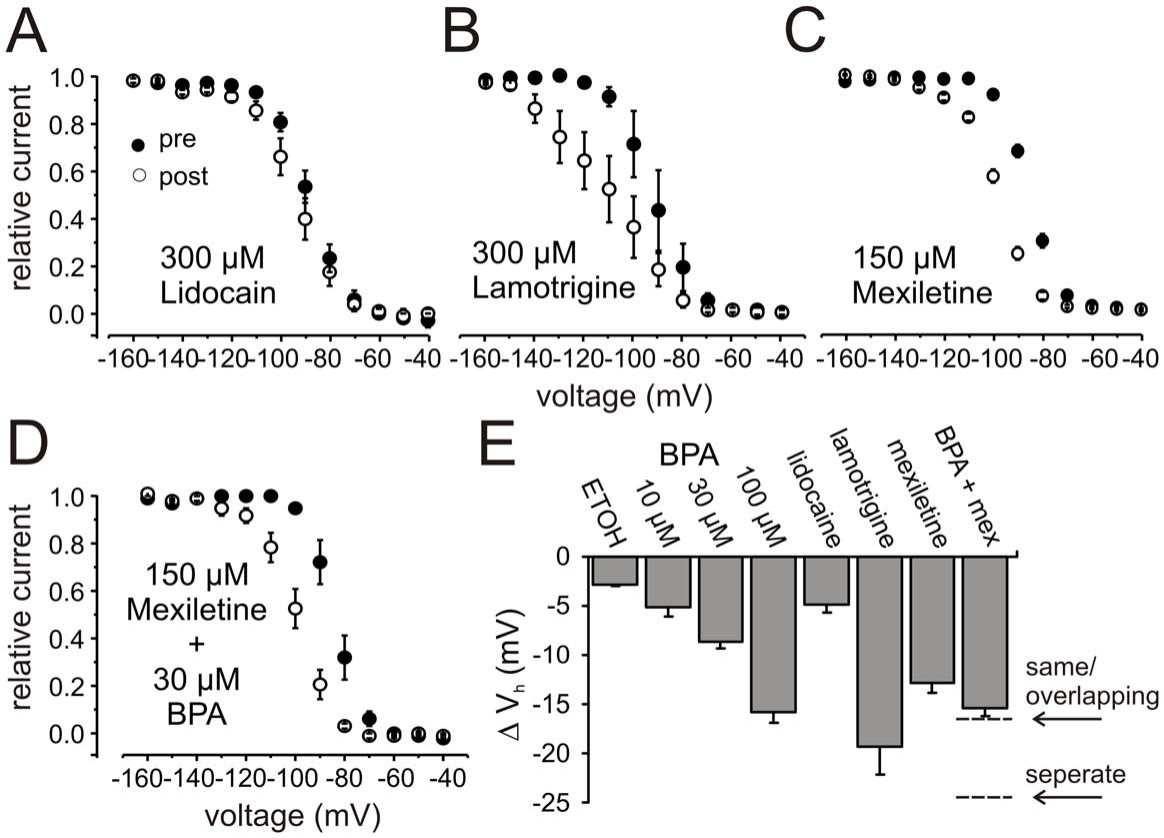
Effects of local anesthetics on steady-state fast inactivation. Steady-state fast inactivation of hNav1.5 before and after application of 300 µM lidocaine (A, n = 9), 300 µM lamotrigine (B, n = 4), 150 µM mexiletine (C, n = 10), and co-application of 150 µM mexiletine and 30 µM BPA (D, n = 4). For protocol see inset in Figure 1C. E: Shift of the midpoints of steady-state fast inactivation of hNav1.5 induced by (from left to right): 0.1% ETOH, 10 µM, 30 µM and 100 µM BPA, 300 µM lidocaine, 300 µM lamotrigine, 150 µM mexiletine and co-application of 30 µM BPA with 150 µM mexiletine. Broken lines indicate predictions using the equations from Kuo et al. [18] for a shift of the midpoints of steady-state fast inactivation following application of a combination of 30 µM BPA and 150 µM mexiletine for the two cases that both drugs bind to separate or the same/overlapping binding site. Our results indicate that BPA and mexiletine share at least parts of the same binding site.

**Figure 3:**
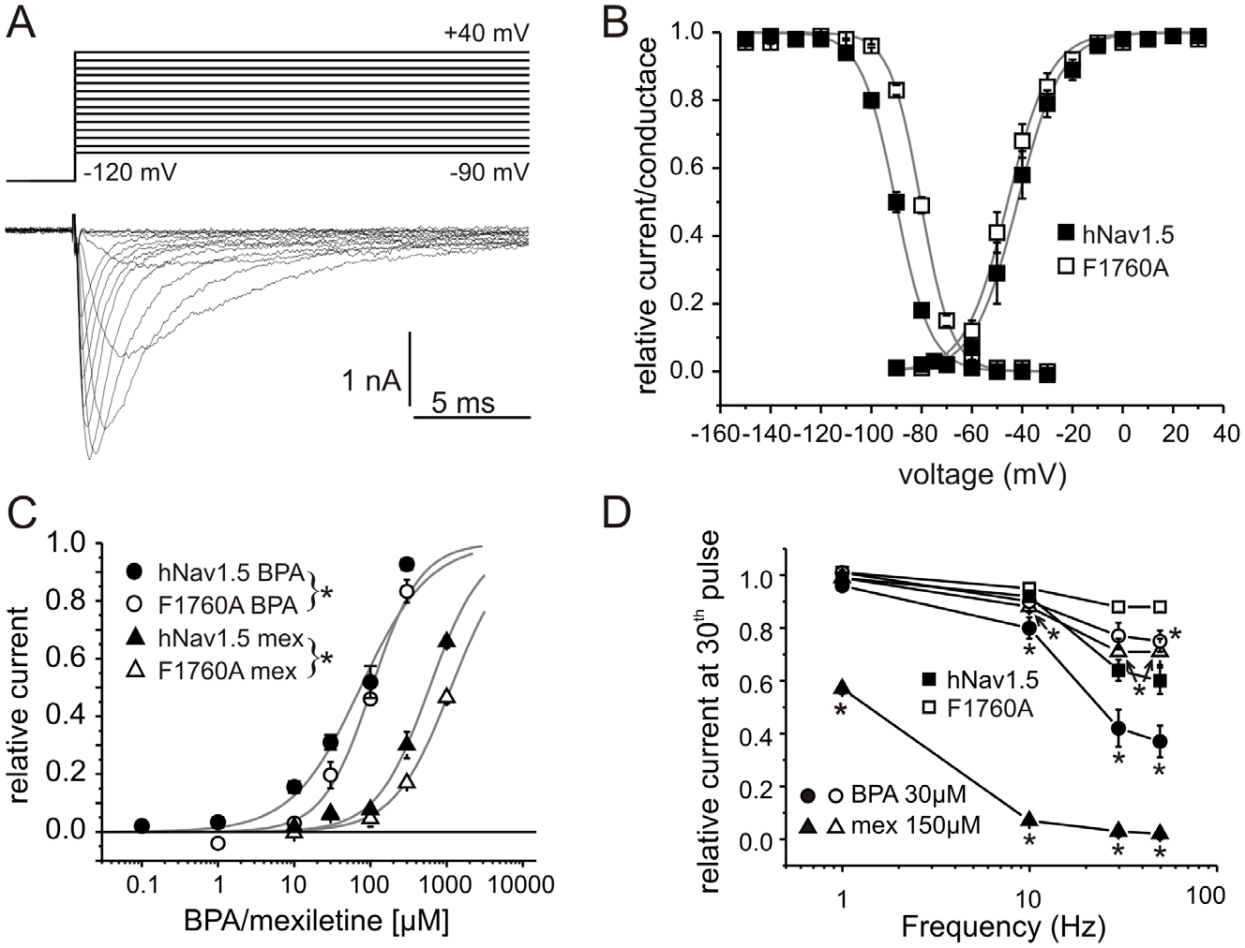
BPA and mexiletine share an overlapping binding site. A: Representative current traces of the mutant channel F1760A evoked by stepping the membrane to the indicated potentials. B: Activation and steady-state fast inactivation of WT hNav1.5 (filled squares, n = 10 and 44, respectively) and the mutant F1760A (open squares, n = 14 and 61, respectively). C: Tonic block of WT hNav1.5 (filled symbols) and the mutant F1760A (open symbols) by BPA (circles) and mexiletine (triangles) at a holding potential of −120 mV, tested every 5 s, n = 3−15 (for protocol see Fig. 1A). Straight lines represent results of a Hill fit, with the following K_d_s: hNav1.5 BPA: 74.0±7.6 µM; F1760A BPA: 104.1±6.7 µM; hNav1.5 mexiletine: 595.9±41.8 µM; F1750A mexiletine: 1142.5±94.0 µM. The mutation significantly reduced the blocking effects of mexiletine and BPA on hNav1.5. The posthoc test revealed a significant difference for all tested concentrations of BPA and for the highest two concentrations of mexiletine. D: Repetitive pulsing of F1760A expressing HEK cells induces almost no use-dependent current decline (open symbols), which can be enhanced to a limited extent by mexiletine (150 µM, open triangles) or BPA (30µM, open circles). In this Figure data for WT hNav1.5 are the same as in Fig. 1B and are included for comparison (filled symbols). Squares represent use dependencies of the channel without drug application. Asterisks indicate significant differences compared to the untreated channel (WT, F1760A) for each frequency, respectively.

100 µM BPA induces a left shift of the voltage-dependence of steady-state fast inactivation by 15.8 mV, suggesting that it stabilizes the fast inactivated state (Fig. 1C). LAs induce a shift of steady-state fast inactivation to more hyperpolarized potentials and prefer to bind to the inactivated state of the sodium channels (e.g. [13], [27]), comparable to the BPA effect. We therefore compared the effect of BPA on steady-state fast inactivation with those of the LAs mexiletine, lidocaine and lamotrigine. As BPA was dissolved in ethanol, 0.1% ETOH was tested as vehicle, which induces a small hyperpolarizing shift in V_half_ of steady-state fast inactivation itself, but this shift is smaller than those found for BPA or the tested LAs. For all LAs, a higher concentration than for BPA is needed to attain comparable effects on steady-state fast inactivation (Fig. 2). For example, 150 µM mexiletine induced a 12.7±1.2 mV (n = 11) hyperpolarizing shift (Fig. 2C), whereas only 100 µM BPA is sufficient for a 15.8±1.1 mV (n = 9) shift in the same direction (see Fig. 2E for comparison). BPA and LAs share two prominent characteristics: both stabilize the inactivated state and induce a use-dependent block when applied to hNav1.5-expressing HEK cells (Fig. 2A). These two similarities suggest that both substances might share a common binding site in hNav1.5. If that is the case, then co-application of mexiletine and BPA should result in a predictable shift of V_half_ of steady-state fast inactivation when the corresponding K_r_s and K_i_s are known (see Methods [17], [18]). With our experimental data we determined K_r_ and K_i_ for BPA (30µM) as: K_r_ = 58.6±8 µM > K_i_ = 8.2±2.4 µM, and for mexiletine (150µM): K_r_ = 606.2±127.5 µM >> K_i_ = 6.6±1.9 µM. 100 µM as well as 30 µM BPA are sufficient to induce a hyperpolarizing shift of steady-state fast inactivation (Fig. 2E) and the low concentration of 30 µM BPA displayed a prominent tonic block (see Fig. 1A). The shift of V_half_ induced by 30 µM BPA is clearly larger than that induced by 0.1% ethanol itself (Fig. 2E) and was therefore chosen as concentration for the following experiments. This concentration corresponds to approximately half the K_d_ for tonic block (74.0±7.6 µM, Fig. 1A). Correspondingly, we chose 150 µM mexiletine, as this is a concentration that displays both, use-dependent and a small tonic block (Fig. 1B and 3C), ensuring that this concentration is high enough to have the drug bound with its binding site.

The broken lines in Fig. 2E indicate the predicted shift in V_half_ following application of 30 µM BPA, 150 µM mexiletine and a combination of both for the two cases where a separate versus single or overlapping binding site exists. As shown in Fig. 2E, our experimental data predict that BPA and mexiletine share at least parts of the same binding site.

### BPA Binding Site Overlaps with that of Mexiletine

The F1760A mutation of hNav1.5 lies within the known LA receptor site in the S6 segment of domain IV [12]. Activation of F1760A-mutated Nav1.5 was not altered compared to WT, but steady-state fast inactivation was shifted to more depolarized potentials by 10.5 mV (Fig. 3, V_half_/*k*: WT: −90.4±0.8 mV/5.1±0.1 mV, n = 51; F1760A: −79.9±0.6 mV/5.8±0.1 mV, n = 61), as reported previously [12]. F1760A displayed a reduced tonic and use-dependent block compared to WT hNav1.5 when 150 µM mexiletine was applied, indicating interference with the binding site of mexiletine on hNav1.5 (Fig. 3C, D, squares and triangles).

Tonic block of BPA was reduced by the F1760A mutation in comparison with WT hNav1.5 (Fig. 3C), demonstrating the importance of this residue for BPA binding. Similarly, the use-dependent block of 30 µM BPA was robustly reduced at almost all frequencies tested, strongly suggesting that mexiletine and BPA at least partly share a common binding site (Fig. 3D).

### Homology Model of hNav1.5 and Docking Predictions for BPA and Mexiletine

Most Na_v_ closed-state models reported in the literature (e.g. [28]) were developed using the KcsA potassium channel crystal structure [29] to produce homology models. However, the recently determined crystal structure of the bacterial sodium channel NavAB [16] provides a more suitable template. One consequence of using this alternative template is that the resulting Na_v_ pore region has considerably greater internal volume in comparison with our previous closed-state model based on KcsA [28]: 95 versus 45 water molecules could be accommodated in the pore. The pore fenestrations in particular contribute to the greater spaciousness.

**Figure 4:**
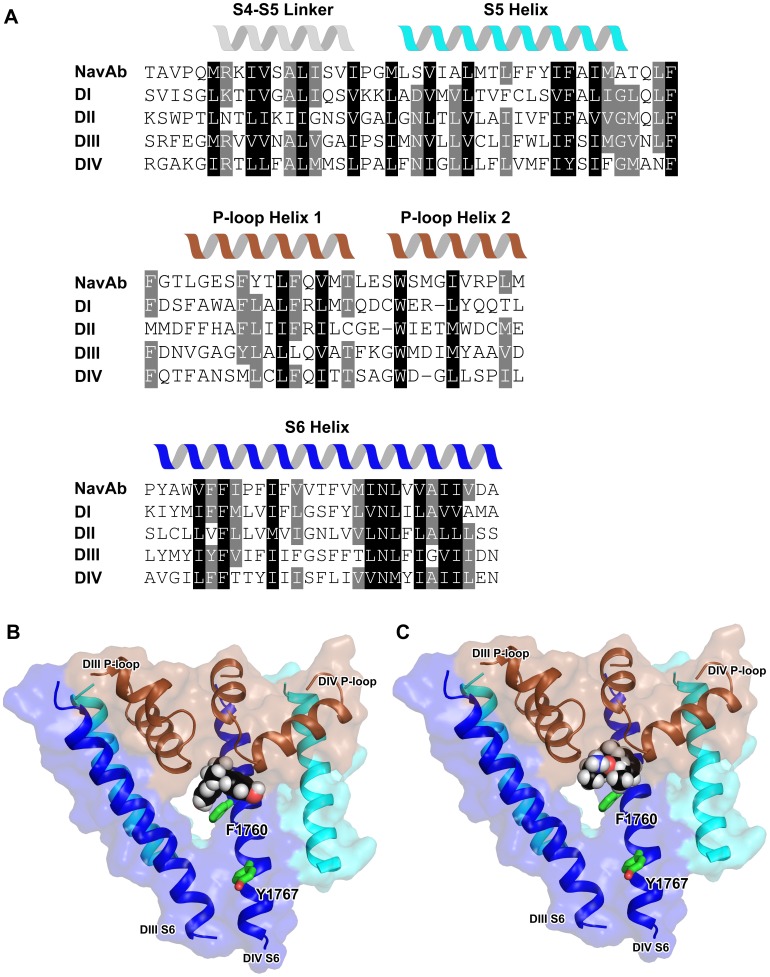
Modeling and ligand docking. A: Sequence alignment of the pore regions of NavAb and hNav1.5. Helical regions in the NavAb structure are indicated by the cartoons above the sequence. Residues shaded in black by sequence show high sequence homology, those in grey display moderate homology. B–C: Molecular model for the hNav1.5 structure. The protein structure is shown in ribbon mode and the ligands [(B) BPA and (C) mexiletine] are shown in space-filling mode. The estimated free-energy of binding is −6.12 kcal/mol for BPA and −4.11 kcal/mol for mexiletine. Selected channel residue side chains are depicted as green sticks and the accessible surface is shown with 70% transparency. Domains I and II are not displayed to aid visualization.

We undertook docking predictions to identify a potential binding site for BPA. These studies suggested that this compound is capable of binding in a cavity adjacent to the channel selectivity filter (Fig. 4B). BPA makes contact with residues on the P-loops of DIII (T1417) and DIV (T1708, S1710) and the DIV S6 (I1756, I1757, S1759, V1763). However, the most extensive interaction occurs with F1760 of DIV S6, the side chain of which projects into the pore lumen. Both phenol rings of BPA contact F1760 and consequently the compound effectively straddles this side chain. In contrast, the Y1767 side chain located closer to the cytoplasmic entrance of the pore is distal from BPA. Y1767 plays an important role in use-dependent inhibition of LAs but has no significant impact on tonic block when mutated [30].

The most energetically-favorable docking prediction of mexiletine in the vicinity of F1760 positioned this compound in the same binding pocket as BPA (Fig. 4C). Mexiletine’s amine group is not in contact with the F1760 ring but instead comes into close proximity to the selectivity filter. The hydroxyl group of the S1759 residue on DIV S6 is <4 Å away from the LA molecule and has potential for hydrogen bond formation. The aromatic ring of mexiletine makes a hydrophobic contact with I1756 (hidden behind the ligand in Fig. 4C), a residue that was found to negatively affect tonic block – but not use-dependent block – when mutated [12]. As with BPA, mexiletine does not contact the Y1767 side chain, as they are located >12 Å apart.

### SMD Simulations of Ligand Ingression

**Figure 5:**
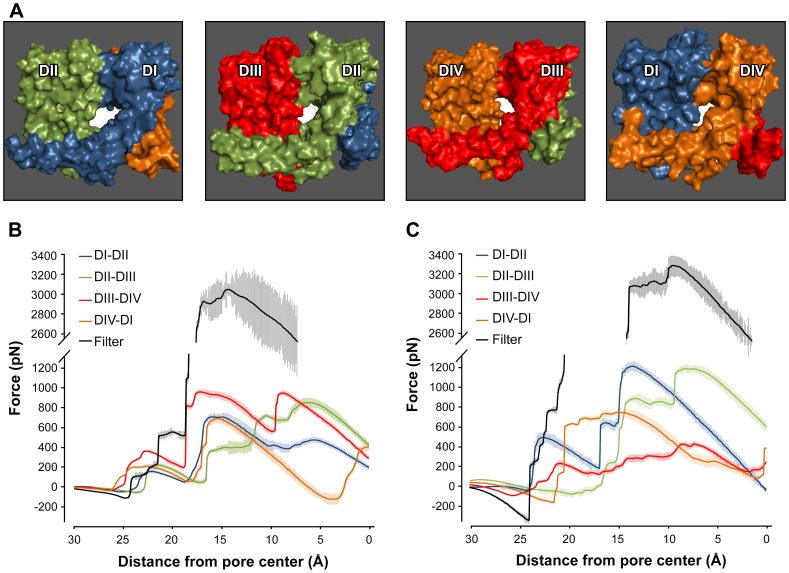
Side fenestrations offer a new entrance pathway. A: Surface representations of hNav1.5 side fenestrations as viewed from the membrane. The model is colored by domain with the central white region showing the fenestration located at the interface of each domain pair. B–C: Force exerted on the center-of-mass of BPA (B) or mexiletine (C) during steered molecular dynamics simulations. Each trace is the averaged force from 21 simulations displayed as mean ± SEM.

Both BPA and mexiletine display a prominent tonic block indicating that the compounds gain access to the closed-state pore. As the intracellular face of the pore is tightly occluded in the closed conformation, the ligands must gain access via an alternative route: either the selectivity filter or the side fenestrations. Constant-velocity SMD simulations were undertaken to assess and compare possible routes for BPA and mexiletine ingress. In these studies the ligand is considered to be attached by a virtual spring to a point that moves through space at constant velocity. If the ligand encounters an impediment to movement, the spring stretches and the resultant force can be calculated. The four pore fenestrations (Fig. 5A) and the selectivity filter of the hNav1.5 model represent constriction points of different shape and dimensions and therefore present different energetic barriers to a mobile ligand, which may have to displace side-chains and possibly backbone atoms of residues lining the pathway in order to traverse.

Fig. 5B and C show the calculated forces exerted on BPA or mexiletine as the ligand progresses from exterior to interior of the channel pore via different routes. Both BPA and mexiletine require the application of an extensive force (>3,000 pN) to permit their transit through the pathway involving the tightly-constricted selectivity filter (Fig. 5B/C, black traces). In stark contrast, a force of ∼700–900 pN for BPA and of ∼450–1200 pN for mexiletine was sufficient to navigate the fenestrations. The force profiles for each fenestration differ, which reflects the pseudosymmetry of the Nav1.5 structure. BPA traverses two of the four fenestrations with apparent ease compared to mexiletine (DI–DII and DII–DIII: ∼700 and ∼850 pN for BPA; >1200 pN for mexiletine, respectively). Both compounds encounter a similar resistance when traversing DIV–DI side opening (∼700 pN), and mexiletine needs less maximal force than BPA for only one entrance path, the side fenestration between DIII and DIV (BPA: ∼950 pN; mexiletine: ∼450 pN). The differences derive from dissimilar flexibility and lateral movement of the compounds through the fenestration (see video S1, S2, S3, S4).

## Discussion

With this study we have identified for the first time a cardiac target for BPA: the compound blocks currents through hNav1.5 in a tonic- and use-dependent manner. The modulation profiles of hNav1.5 by BPA and LAs share striking similarities. Both ligand types have a higher affinity for the inactivated state (K_r_>K_i_), display a shift of steady-state fast inactivation to more hyperpolarized potentials and, in addition to a strong tonic component, both show a strengthening of use-dependent current decline. The effect on steady-state fast inactivation of co-applied BPA and mexiletine (Fig. 2) was not additive; the observed shift instead indicated competitive binding. To test the hypothesis that the compounds exert their modulation via interaction with a common receptor site, BPA and mexiletine were tested with the F1760A mutant. This single substitution greatly attenuated both use-dependent and tonic block by BPA (Fig. 3), thus marking another commonality with LAs [12], [14], [31], [32], [33] and supporting the prediction of an overlapping binding site in the pore.

F1760 is a critical binding determinant for LAs and engages pore-blockers in two distinct interactions depending on the functional state of Na_v_. For use-dependent block of the open-inactivated pore, an aromatic side-chain is required at the F1760 position to satisfy a putative cation-π interaction with the tertiary amine of a LA [12], [14], [15]. However, BPA lacks a charge and its interaction with the open-inactivated pore may therefore share similarities with LAs that are neutral at physiological pH e.g. benzocaine, phenytoin or carbamazepine [31], [34]. For tonic block, the physicochemical requisite is hydrophobicity – not aromaticity – for interaction at the F1760 position [30], which suggests an alteration in ligand contacts between the open- and closed-state pore in accordance with the modulated receptor hypothesis [13].

The K_i_s for mexiletine and BPA are almost the same although mexiletine displays a larger apparent use-dependent block. This may be due to the higher solubility of mexiletine in water, which would allow quicker access to the binding site from the intracellular side when the channel is opening repetitively. K_i_, on the other hand, was determined after the cells were held on an inactivating potential for several seconds, rendering time-dependent processes less relevant. 30 µM BPA and 30 µM mexiletine both induce significant use-dependent block (Fig. 1B), whereas tonic block with 30 µM mexiletine is almost absent. This also suggests that BPA accesses the binding site in the closed-state channel with comparatively greater ease than mexiletine.

The availability of a bacterial sodium channel crystal structure [16] has enabled us to examine the structural basis of tonic block using a hNav1.5 homology model. Automated docking predictions identified a common binding site for both BPA and mexiletine in a hydrophobic cavity atop the F1760 side chain on S6 of domain IV (Fig. 4B–C). This pronounced cavity was not evident in a previous closed-state Na**_v_** model based on the KcsA potassium channel crystal structure [28]. F1760 forms significant van der Waals contact with both docked ligands and, in agreement with our electrophysiology data, its mutation to the small side-chain alanine residue would diminish a key underpinning contact. It is the hydrophobic section and not the amine group of mexiletine that contacts F1760, which precludes a cation-π-interaction and is consistent with the hydrophobic but not aromatic requirement at this position for tonic block [30]. Therefore this tonic block receptor is distinct from the use-dependent-block binding site for which residue Y1767 was shown to be important [30]. Y1767 does not appear to form part of the BPA binding site. Significant structural rearrangements would be necessary to reposition a LA to form a cation-π interaction with F1760 and simultaneously contact Y1767 (e.g. a change in the F1760 side-chain rotamer, [12]). In the structural model Y1767 is closer to the cytoplasmic pore opening and may represent the first major binding determinant for substances entering the pore from the intracellular side (use-dependent block). F1760 on the other hand is closer to the side fenestrations, offering a possible binding site for molecules potentially entering via this pathway (tonic block).

BPA is a potent tonic blocker (K_r_ = 58.6±8 µM) and its affinity for the closed-state pore may derive from its considerable hydrophobicity (logP 4.04; Table S1). The docking predictions identify I1756 as a key hydrophobic contributor to the tonic-block receptor (Fig. 4B–C, located behind the ligands). Mutation of I1756 to alanine produces a modified tonic block, not necessarily due to altered binding, but it seems to ease access to the pore for extracellularly-applied LAs [12], [35], [36]. Permanently-charged derivatives of lidocaine are unable to partition completely in the membrane but can still access the closed-state pore of the I1756A mutant. Studies with µ-conotoxin rule out the selectivity filter as the route for this novel entry pathway [36]. I1756 is located in the model in the vicinity of the predicted interface between the lipid bilayer, extracellular solution and the DIII–DIV side fenestration. It is possible that substitution to the small side chain alanine enlarges the DIII–DIV side fenestration, such that a charged LA molecule which is semi-partitioned in the lipid bilayer and orientated with the tertiary amine contacting negatively-charged lipid head group(s) may diffuse laterally into the pore, passing I1756A.

We employed the hNav1.5 model to qualitative assess potential routes for BPA or mexiletine ingress using SMD simulations. The force exerted on the mobile ligand during the simulations was used as a descriptor of how well the ligand would fit through the access pathway (Fig. 5B–C). The highly-constricted selectivity filter comprised the path of greatest resistance for both ligands while the pore fenestrations were comparably more accessible. The four fenestrations hindered the crossing of BPA to a similar extent (peaks of ∼700–900 pN in the force profiles; Fig. 5B) but in the case of the more flexible mexiletine the fenestration at the DIII–DIV interface proved most accessible (peak of ∼425 pN), whereas DI–DII and DII–III showed a higher resistance to mexiletine than to BPA entry. A ligand ingressing the pore via the DIII–DIV fenestration would directly contact F1760 and therefore be in favorable position to occupy the tonic-block receptor. The compatibility of the DIII–DIV fenestration with closed-state ligand ingress when compared with the selectivity filter correlates with results from an analogous study by Bruhova et al. [37]. However, this latter study used the KcsA potassium channel structure as template and consequently the pore fenestrations of the Na_v_ homology model were significantly smaller.

BPA exposure has recently been linked to heart disease [7], [8], raising the question of a cardiac target. The highest reported blood level of BPA is 290 nM [38], which is still lower than 1 µM, the lowest concentration to have a significant effect on hNav1.5 in our study (Fig. 1A). The lowest concentration tested at which hNav1.5 currents were not affected was 100 nM (no effect level, NOEL). The mean human blood levels are reported to be ∼10 nM [39], just within the tolerable range, according to safety calculations based on these results. There might be a very slight risk that BPA may have adverse effects on cardiac function, especially if patients are preconditioned (e.g. treated with class 1 antiarrhythics). Still, arrhythmias induced by BPA via its effects on Nav1.5 alone do not seem very likely. Our recordings were performed in a heterologous expression system at room temperature, and many factors may influence potential cardiac BPA effects in physiological conditions, such as additional binding proteins and body temperature. We have determined a short term effect of BPA, and potential long term effects of BPA exposure to cardiac function remain to be investigated.

BPA was shown to affect voltage-gated sodium currents in dissociated dorsal root ganglion neurons [40]. However, these studies did not investigate BPA action in a defined heterologous expression system or identify a specific binding site.

In this study, we have identified the first cardiac-specific target for BPA and further localized its binding site to the LA receptor in the hNav1.5 pore. Modeling and computational methods provide a basis for understanding BPA-binding interactions in the closed-state channel and assess routes for entry into the pore lumen. Our data may contribute to the on-going debate on BPA safety limits at a time of heightened public concern about BPA exposure.

## Supporting Information

Table S1 **Physicochemical characteristics of BPA and local anesthetics.**
(DOCX)

Video S1 **Steered molecular dynamics study of mexiletine entering the pore of the homology model of Nav1.5 via the side fenestration DIII–IV.**
(MPG)

Video S2 **Steered molecular dynamics study of BPA entering the pore of the homology model of Nav1.5 via the side fenestration DIII–IV.**
(MPG)

Video S3 **Steered molecular dynamics study of mexiletine entering the pore of the homology model of Nav1.5 via the selectivity filter.**
(MPG)

Video S4 **Steered molecular dynamics study of BPA entering the pore of the homology model of Nav1.5 via the selectivity filter.**
(MPG)
