# Supplementary material for: Bisphenol A Binds to the Local Anesthetic Receptor Site to Block the Human Cardiac Sodium Channel
Source: PLoS One. 2012 Jul 27;7(7):e41667. doi: 10.1371/journal.pone.0041667 (PMC3407203; doi:10.1371/journal.pone.0041667)
Supplement: Table S1 — Physicochemical characteristics of BPA and local anesthetics. (DOCX) [file pone.0041667.s001.docx]

## Supplementary Material:

**Supplementary Table S1.** Physiochemical characteristics of BPA and local anesthetics

|  | **MW (g/mol)** | **Basic pKa** | **N(pKa)** | **LogP** | **LogD at pH 7.3** |
| --- | --- | --- | --- | --- | --- |
| **BPA** | 228.29 | 0 | 1 | 4.04 | 4.04 |
| **Mexiletine** | 179.26 | 9.52 | 0.006 | 2.46 | 0.3 |
| **Lidocaine** | 234.34 | 7.75 | 0.262 | 2.84 | 2.26 |
| **Lamotrigine** | 256.09 | 5.87 | 0.964 | 1.93 | 1.91 |
| **Benzocaine** | 165.22 | 2.78 | 0.999 | 1.5 | 1.5 |

Values were calculated using Marvin 5.7 software from ChemAxon (Budapest, Hungary). The neutral fraction of ligand at pH 7.3 was calculated using the Henderson-Hasselbalch equation: N(pKa) = 10^pH^/(10^pH^+10^pKa^).

**Supplementary Videos S1-S4.** Steered molecular dynamics studies of mexiletine and BPA entering the pore of the homology model of Nav1.5 via the side fenestration DIII-IV or the selectivity filter.
